# Satisfaction of Primary Healthcare Dentists after the Completion of a Distance Learning Course in Pediatric Dentistry

**Published:** 2019-08

**Authors:** Caren Serra BAVARESCO, Silvana BRAGANÇA, Francine de Paula FRIES, Giordano Santana SÓRIA, Flávio Renato Reis DE MOURA, Elken Gomes RIVALDO, Otávio Pereira D’AVILA, Roberto Nunes UMPIERRE, Erno HARZHEIM, Jonas Almeida RODRIGUES

**Affiliations:** 1. Discipline of Dentistry and Society, Graduate Program in Dentistry, School of Dentistry, Lutheran University of Brazil, Canoas, RS, Brazil; 2. Discipline of Pediatric Dentistry, Department of Conservative Dentistry, School of Dentistry, Federal University of Rio Grande do Sul, Porto Alegre, RS, Brazil; 3. School of Dentistry, Lutheran University of Brazil, Canoas, RS, Brazil; 4. Graduate Program in Dentistry, School of Dentistry, Lutheran University of Brazil, Canoas, RS, Brazil; 5. Discipline of Epidemiology, Department of Collective Health, School of Dentistry, Federal University of Pelotas, Canoas, RS, Brazil; 6. Discipline of Family and Community Medicine, Department of Family and Community Medicine, School of Medicine, Federal University of Rio Grande do Sul, Porto Alegre, RS, Brazil; 7. Municipal Health Department, Porto Alegre, RS, Brazil

**Keywords:** Pediatric dentistry, Satisfaction, Deciduos teeth, Primary healthcare, Distance learning

## Abstract

**Background:**

The aim of the present study was to assess the level of satisfaction of dentists working in primary healthcare (PHC) with a Distance learning (DL) course in pediatric dentistry offered by the TeleHealthRS center, and to the investigate possible associations between the variables indicative of their satisfaction and their performance on the questionnaires applied before and after the course.

**Methods:**

The course was offered in 2015 by the Federal University of Rio Grande do Sul (UFRGS) together with the TeleHealthRS center in Brazil. Data were collected on the participants’ personal and professional profile, their pre- and post-course knowledge about pediatric dentistry, and their satisfaction with the course. Student’s *t*-test and the ANOVA test were used to assess the possible associations between the variables indicative of their satisfaction and their performance on the questionnaires applied before and after the course.

**Results:**

Overall, the participants were satisfied with the course, however, no statistically significant association was found between the variables indicative of their satisfaction and the grades they earned on the pre- and post-course questionnaires.

**Conclusion:**

The available pediatric dentistry course received positive evaluations from the participants, constituting a possible strategy for the qualification of primary care dentists. Future studies are warranted to further investigate the expectations of DL course participants, aiming to enhance the quality of future editions of this learning modality for pediatric dentistry contents.

## Introduction

The intense renovation of scientific knowledge requires professional updating in order to ensure the quality of health care. Some of the strategies used as a tool for solving doubts and to update knowledge on oral health, considering that distance and the use of technological resources are essential factors, is called teleodontology. Among the most commonly used forms interaction, teleassistance and teleconsulting stand out, as well as the activities related to education called teleducation (1, 2). Teleducation can be understood as conducting conferences, classes and courses at a distance using information and communication technologies (ICTs) (3).

Dentistry was also included in this program, and can thus count on receiving tele-assistance, tele-diagnosis and tele-education services (3). The expansion of access by professionals of the dental health team to materials prepared with technical content based on scientific evidence, through technological resources, is of fundamental importance. With the increasing use of mobile devices and social media, the search for educational resources, based on validated content and easy accessibility, has been consolidated as a new way of learning in the field of health (4).

Distance learning (DL) has become a method increasingly used for continuing education and professional development (1). In this context, the Brazilian Ministry of Health created the Telehealth Program, whose aim is to qualify professionals working within the Family Health Strategy (FHS), and thus strengthen primary healthcare (PHC) actions (2). The first DL evaluation studies were based on experimental designs using qualitative and mixed methods. They included assessments of course completion rates, grades earned by participants before and after taking the course, student attitudes and habits related to technology, and student satisfaction with the course (5).

Some studies carried out in Brazil have shown positive results related to the effectiveness of distance courses. In this context, an e-learning training course on atraumatic restorative treatment (ART) was developed for Brazilian dentists (6). E-learning has the potential of improving the knowledge that dentists working in the public health system have about ART, especially those with less clinical experience and less knowledge about the subject.

However, despite the fact that some studies previously published in the literature, report high rates of student satisfaction with DL courses in the field of dentistry (7–10), many studies do not discuss user assessment as a necessary outcome. In this respect, the aim of the present study was to assess student satisfaction with a DL course in pediatric dentistry offered by the TeleHealthRS center.

## Methods

This was a descriptive, analytical, cross-sectional study based on an analysis of the satisfaction of professionals who completed a DL course in Pediatric Dentistry, and who answered a satisfaction questionnaire prepared by the researchers. The course, titled “Pediatric Dentistry in Primary Healthcare,” (11) was offered in 2015 by the Federal University of Rio Grande do Sul (UFRGS) together with the TeleHealthRS center in Brazil. This study was approved by the Research Ethics Committee at UFRGS (register no. 1.302.271).

The structure of the course was previously described (11). Sampling for the study was conducted in terms of convenience and had, as its target audience, dental surgeons acting at PHC in Brazil who enrolled in the course. The criteria for inclusion and participation in the course were: being a dental surgeon; being with register and duties up-to-date along with the council of his category in the region, and acting at PHC. In order to analyze the data, only the participants who accepted to take part in the study, by means of confirmation in an electronic Informed Consent form, were included. Another important fact is that they needed to have answered the pretest, a quiz about personal and professional profile and the post-test. A pretested questionnaire consisting of 15 questions was used to assess initial dental knowledge of participants. After the course, participants retook the same initial questionnaire. Participant performance on the dental knowledge questionnaire improved pre- to post-test.

To analyze student satisfaction with course were used the questionnaires about the personal and professional profile of participants, about their knowledge of pediatric dentistry before and after the course, and about their evaluation of the course, including different variables indicative of their satisfaction. For the satisfaction analysis, a 5-point Likert scale was used (Very Bad, Bad, Regular, Good, Excellent). The demographic variables collected were age and sex. For the professional profile, type of training institution, time since graduation, years working in PHC, type of training institution and specialization were evaluated.

The descriptive statistics of the students’ satisfaction with the course was expressed by absolute numbers and percentage rates. The variables of the professional and personal profile were grouped into categories, as follows: A) Age: 20–29 yr, 30–39 yr, or +40 yr; B) type of training institution: public or private; C) time since graduation: up to 10 yr, 11–20 yr, or +20 yr; D) years working in PHC: up to 5 yr, 5–10 yr, or +10 yr. Student’s T-test and the ANOVA test were used to assess possible associations between the variables indicative of their satisfaction and their performance on the questionnaires applied before and after the course. A *P*-value<0.05 was considered significant. The statistical analyses were performed using STATA software, version 12.0 (Stata Corp., College Station, TX, USA).

## Results

Overall, 430 participants signed up for the DL course, 15 of denied enrollment for not being dentists, 42, for failing to complete the enrollment stages, and 18, for failing to respond to the pre-course questionnaire in a timely manner. Of the 355 remaining participants, 10 did not agree to participate in the survey, 89 failed to respond to the questionnaire on their personal and professional profile, 34 failed to respond to the pre-course questionnaire, 2 were excluded because their results were lost, and 19 were excluded for other reasons, leaving a final sample of 201 students. Of these, 40 individuals answered the satisfaction questionnaire, corresponding to 19.90% of the sample. However, only 31 participants (15.42%) completed the satisfaction and personal/professional profile questionnaires and were included in the analysis of the association between the grades they earned and their satisfaction with the course. There was a predominance of female participants, older than 30 yr, and with less than 5 yr working in PHC (Table 1).

**Table 1:** Personal and professional profile of students who answered a questionnaire on their satisfaction with the DL course in pediatric dentistry (n = 40)

***Variables***	***N (%)***
Sex	
Male	6 (15)
Female	34 (85)
Age	
20–29 yr	8 (20)
30–39 yr	17 (43)
40+ yr	15 (37)
Holds a specialization degree	
Yes	18 (64)
No	10 (36)
Type of training institution	
Public	18 (64)
Private	10 (36)
Time since graduation	
Up to 10 yr	14 (48)
11–20 yr	9 (31)
20+ yr	6 (21)
Years working in PHC	
Less than 5 yr	15 (53)
5–10 yr	9 (32)
10+ yr	4 (15)

Overall, the participants were satisfied with the course and attributed positive values to the variables evaluated. However, no statistically significant association was found between the variables indicative of student satisfaction and the grades they earned on the pre- and post-course questionnaires (Tables 2 and 3).

**Table 2:** Stratification of the average grades earned by participants on a questionnaire applied prior to a DL course in pediatric dentistry into the different variables indicative of their satisfaction with it (n=31)^*^

***Variables***	***N (%)***	***Mean grade (SD)***	***P-value***
General opinion of the course			
Regular	3 (9.70)	8.22 (1.02)	0.73
Good	13 (41.9)	8.10 (0.81)	
Excellent	15 (48.4)	7.96 (0.99)	
Opinion about the duration of the course			
Regular	2 (6.50)	8.71 (0.95)	0.90
Good	12 (38.7)	9.14 (0.64)	
Excellent	17 (54.8)	8.93 (0.90)	
Teacher’s knowledge			
Good	6 (19.4)	8.11 (0.50)	0.83
Excellent	25 (80.6)	8.02 (0.97)	
Pedagogy			
Regular	1 (3.20)	8.00 (0.00)	0.15
Good	11 (35.5)	7.94 (0.81)	
Excellent	19 (61.3)	8.10 (0.97)	
Suggested Topics			
Good	4 (14.9)	8.00 (1.08)	0.90
Excellent	23 (85.1)	8.05 (0.85)	
Question solving			
No	19 (61.3)	7.96 (0.84)	0.54
Yes	12 (38.7)	8.16 (0.98)	
Opinion about the audio			
Regular	1 (3.30)	8.67 (0.00)	0.51
Good	13 (41.9)	7.84 (0.86)	
Excellent	17 (54.8)	8.15 (0.93)	

* Student’s t-test was used for dichotomous variables, and the ANOVA test was used for polytomous variables.

**Table 3:** Stratification of the average grades earned by participants on a questionnaire applied after a DL course in pediatric dentistry into the different variables indicative of their satisfaction with it (n=31) ^*^

***Variables***	***N (%)***	***Mean grade (SD)***	***P-value***
General opinion of the course			
Regular	3 (9.70)	8.71 (0.00)	0.27
Good	13 (41.9)	9.06 (0.63)	
Excellent	15 (48.4)	9.00 (0.99)	
Opinion about the duration of the course			
Regular	2 (6.50)	8.71 (0.95)	0.70
Good	12 (38.7)	9.14 (0.64)	
Excellent	17 (54.8)	8.93 (0.98)	
Teacher’s knowledge			
Good	6 (19.4)	9.14 (0.53)	0.62
Excellent	25 (80.6)	8.96 (0.85)	
Pedagogy			
Regular	1 (3.20)	8.71 (0.00)	0.76
Good	11 (35.5)	9.18 (0.65)	
Excellent	19 (61.3)	8.91 (0.88)	
Suggested Topics			
Good	4 (14.9)	8.88 (1.08)	0.79
Excellent	23 (85.1)	8.99 (0.85)	
Question solving			
There was none	19 (61.3)	8.98 (0.89)	0.85
Yes	12 (38.7)	9.03 (0.66)	
Opinion about the audio			
Regular	1 (3.30)	8.71 (0.00)	0.89
Good	13 (41.9)	8.95 (0.95)	
Excellent	17 (54.8)	9.05 (0.70)	

* Student’s T-test was used for dichotomous variables, and the ANOVA test was used for polytomous variables.

## Discussion

Technological innovation has had a major impact on the educational system, increasing its visibility and accessibility, especially in communities farther away from large centers. In addition, DL allows users of the system to participate in educational activities at the times and in the locations most convenient to them (12). This type of strategy is particularly relevant for the training of professionals working in the primary healthcare public network, limited time in their work schedules to attend face-to-face courses offered in places far from their job locations (13).

The results of the present study demonstrated high student satisfaction rates with the DL strategy, confirming the other reported results (14, 15). On the other hand, no association was found between student satisfaction and the grades earned on the questionnaires applied before and after the course, suggesting that there is no direct relationship between the degree of student satisfaction and the knowledge acquired during the course or held previously on the subject. Only a small portion of those enrolled in the course answered the satisfaction questionnaire, considered a bias of the present study. Only students most satisfied with the course submitted their perception about it, the study’s positive data may have been overestimated.

Another aspect associated with the level of student satisfaction with DL resources is student familiarity with information technology and digital platforms. Accordingly, the need to train professionals on how to use digital communication strategies should be considered, since difficulties in manipulating available resources can limit access and negatively impact user satisfaction with this tool (2).

However, studies in the literature mention rates of return of online questionnaires in the range of 14% to 55.2% (16–18), with an average of 37.9%, the response rate presented in the present study (15.49%) may constitute a bias in the observed data and should be interpreted with caution. Although the electronic method is more agile and practical, both for the participants and for the researchers, the commitment to their response may be reduced, related to their impersonality.

In addition, despite the growing access to DL dentistry courses in the last decade, their effectiveness in the public health area still needs to be further investigated with studies having more robust designs.

## Conclusion

The DL course in pediatric dentistry offered by the TeleHealthRS center met the expectations of those enrolled in it; however, no associations were found between the degree of participant satisfaction and the grades earned in the evaluations made of the knowledge acquired during the course. Future studies are warranted to further investigate the expectations of DL course participants, aiming to enhance the quality of future editions of this learning modality for pediatric dentistry contents.

## Ethical considerations

Ethical issues (Including plagiarism, informed consent, misconduct, data fabrication and/or falsification, double publication and/or submission, redundancy, etc.) have been completely observed by the authors.
